# A Treatise on the Diseases of the Eye

**Published:** 1884-10

**Authors:** 


A TREATISE ON THE DISEASES OF THE EYE. *By* J. SOELBERG WELLS, F. R. C. S. *Doctor of Medicine of the University of Edinburgh; Professor of Ophthalmology in King's College, London, Etc., Etc. Fourth American from the Third English Edition. With copious additions, by* CHARLES STEDMAN BULL, A. M., M. D. *Lecturer on Ophthalmology in the Bellevue Hospital Medical College, Etc., Etc. Cloth, pp. 846. Philadelphia:* HENRY C. LEA'S SON & CO.

The salient features of this work are too well known to physicians and students to require more than a cursory survey of its contents. Medicine has kept stride with her sister sciences in adding to her lore and in the practical application of the great researches elicited by the spirit of the age—progress. In no branch of medicine is this more strikingly shown than in ophthalmology. Although not more than three years have elapsed since the publication of the third American edition, the author has felt the necessity of adding several chapters and copious notes to bring it up to our present stage of advancement. The latest researches are either directly mentioned, or, where the subject seems to call for a more detailed account than can be alloted to the narrow frame of a text-book, references are made to articles bearing on the questions involved. The chapters oń sympathetic ophthalmia and glaucoma are singularly complete in this respect, treating, as they do, of the valuable and but recent labors of Alt, De Wacker, and especially Mauthner; also of the inflammatory and neurotic theroids expounded by Kneis, Ad. Weber, Schnabel, Brailey, Mauthner, and Brixtley Smith. Embodied in the chapter on trachoma we find a concise statement of Sattler's germ theory. The author, in his preface, deplores his inability to include in the same chapter the treatment by jequerity, which was brought to public notice whilst the book was in press. The work can be recommended to students and physicians as a text-book superior to any published in the English language. Dentists will find it a repository of the latest and most important data pertaining to their specialty. To those not conversant with foreign languages, who must depend upon fragmentary knowledge gleaned from reviews, will this work be especially welcome. The book is well printed; the numerous illustrations are well executed.

